# Association of tobacco use with the tobacco-related built environment: an ecological study from urban slums of Bhopal, India

**DOI:** 10.1186/s41256-023-00287-1

**Published:** 2023-02-10

**Authors:** Yogesh Damodar Sabde, Vikas Yadav, Abhijit P. Pakhare, Sanjeev Kumar, Ankur Joshi, Rajnish Joshi

**Affiliations:** 1 Environmental Health and Epidemiology, ICMR- National Institute of Research in Environmental Health (ICMR-NIREH), Bhopal, MP 462030 India; 2 Department of Community and Family Medicine, All India Institute of Medical Sciences, Bhopal, MP 462020 India; 3 Department of Medicine, All India Institute of Medical Sciences, Bhopal, MP 462020 India

**Keywords:** Tobacco use, Built environment, Network analysis, Service area, India, Neighbourhood, GIS, ArcMap, QGIS

## Abstract

**Introduction:**

Tobacco is one of the biggest public health problems and a major risk factor for various non-communicable diseases (NCDs). An important aspect of tobacco control strategy could include modifications in the tobacco-related built environment. This study investigated the association between tobacco shop density and tobacco use prevalence in the urban slums of Bhopal city, India.

**Methods:**

We conducted a cross-sectional survey to obtain the distribution of tobacco-related built environment (tobacco shops) in the neighbourhood (400-m service area) of 32 urban slum clusters of Bhopal. We plotted this distribution using the 'network service area analysis' in ArcMap 10.7.1 software. Then, we used an ecological design to determine the association between tobacco shop density and tobacco use prevalence in these 32 clusters (N = 6214 adult inhabitants). We used multiple linear regression analysis to estimate the regression coefficient (adjusted for socio-demographic variables) between tobacco use and tobacco shop density at the cluster level.

**Results:**

The prevalence of tobacco use among all 32 slum clusters ranged from 22.1 to 59.6% (median 40.9% with IQR 31.8–44.2). There were 194 tobacco shops situated in the neighbourhood of all clusters. The median density of tobacco shops was 59.40/km^2^ (IQR 39.9–108.1/km^2^) in the neighbourhoods of slum clusters. Tobacco use prevalence was significantly associated with tobacco shop density (estimate or B = 0.071, *p* value = 0.002) after adjusting for age, literacy, wealth index, and gender ratio.

**Conclusions:**

Tobacco use prevalence is significantly associated with tobacco shop density in the slums of Bhopal city in central India. We need to develop appropriate built environment interventions to control rampant tobacco use.

## Introduction

The world is currently facing a tobacco-use epidemic (1.3 billion tobacco users) that kills over 8 million people annually [1]. Out of this, approximately 7 million deaths result from direct tobacco use, and the remaining million deaths occur due to second-hand exposure to tobacco smoke [2]. Over 80% of tobacco users live in low- and middle-income countries, where the existing health infrastructure is too fragile to handle the additional health burden associated with tobacco use [2]. India is currently the second-largest producer and consumer of tobacco [3]. Global Adult Tobacco Survey (GATS) 2016–2017 reported that there were 267 million adult (15 years and above) tobacco users in India, accounting for 29% of the country’s total adult population [4]. Due to this colossal number of tobacco users, India witnesses 1.35 million deaths yearly [5].

Tobacco consumption is a known risk factor for multiple non-communicable diseases, including various cancers, lung diseases, cardiovascular diseases and stroke [2, 6, 7]. The consequences of tobacco use are not only limited to disabilities and deaths, but it also has innumerable social and economic implications. For example, tobacco use can push families towards poverty by diverting family expenditure on basic needs (i.e., food, shelter, education, etc.) to purchasing tobacco and managing associated health ailments [8, 9]. According to an estimate by John et al., India's economic burden in the year 2017–2018 due to mortality and morbidity among the population aged 35 years and above attributable to tobacco use was USD 27.5 billion, which is nearly 1% of the total GDP of India [10].

In 2003, the World Health Assembly adopted the WHO Framework Convention on Tobacco Control (WHO-FCTC), an evidence-based international treaty to curb the tobacco use epidemic [11]. As one of this convention's signatories, India promulgated the Cigarettes and Other Tobacco Products (Prohibition of Advertisement and Regulation of Trade and Commerce, Production, Supply and Distribution) Act in 2003 (COTPA, 2003). This act laid down various mechanisms to curb the tobacco menace, such as the prohibition of tobacco sale within 100 yards of schools, a ban on tobacco smoking in public places, a restriction on direct or indirect advertisements, an obligation to include pictorial warnings on packets of tobacco products, etc. [12].

Despite global efforts to curb tobacco use, various tobacco products are widely sold throughout South and East Asia. For example, the use of 'betel quid' is quite prevalent in this part of the world for its stimulant effect. Chewing of betel quid is a smokeless form of tobacco use. It comprises of areca nut, tobacco, lime, and Acacia catechu (Katha) wrapped in a betel leaf. It is frequently consumed with other added components like fennel seed, cardamom, clove and other local fragrances (herbal and chemical). Betel quid is conventionally sold at 'tobacco shops', standalone outlets engaged in preparing and selling betel quid. In addition, tobacco shops also sell other tobacco products such as raw tobacco, cigarettes, bidis (hand-rolled cigarettes made of tobacco wrapped in the leaf of Diospyros melanoxylon, a plant native to Asia) and hookah [4]. Due to the low investment cost and high demand for these products, tobacco shops pop up like mushrooms in all habitations of this region [13]. These tobacco shops are an important component of the 'tobacco-related built environment' in a neighbourhood.

According to the published literature, a neighbourhood area (or residential area) ranges from 100 m (metres) to 1600 m (~ 1 mile) from the respondent's house [14]. The neighbourhood includes built environment attributes such as the streets, shops, places of worship, recreational and green spaces, and the transport system [15–17]. The density and distribution of built environment attributes in the neighbourhood influence health and quality of human life [18–21]. In addition, the tobacco-related built environment also affects tobacco use by reducing travel costs and increasing exposure to marketing [19, 22–29]. Therefore, previous research recommended improving the built environment to control tobacco use and achieve tobacco-related endgame goals [30, 31].

Most studies that documented the association between the density of tobacco shops and tobacco use prevalence were conducted in developed countries using secondary data [23]. Moreover, the characteristics of the tobacco-related built environment vary from country to country. Therefore, country-specific data on the tobacco-related built environment is essential to form tobacco control strategies. In the Indian context, such data on the relationship between tobacco shop density and tobacco use prevalence are scarce. Moreover, the only reported study from India (Rath et al.) could not find a clear association between tobacco store density in the community and tobacco consumption [19].

To overcome the limitations of available literature, we have conducted the present research using original data, a larger sample size and modern Geographic Information System (GIS) applications (service area estimation by network analysis). The neighbourhood built environment has a higher impact on the health of people residing in slums as compared to the non-slum population [32, 33]. Therefore, we conducted this ecological study with the objectives to characterise tobacco shops and to investigate the association between tobacco use and tobacco shop density in the slum neighbourhoods of Bhopal city, located in Central India.

## Methods

### Setting

Bhopal is one of the largest cities in the central Indian province of Madhya Pradesh, with a population of around 1.80 million [34]. Bhopal city consists of 14 zones, 85 wards (administrative units of the local governing body, i.e. the Bhopal Municipal Corporation), and over 389 designated urban slums [35]. Slums are defined by the Registrar General of India (Census India 2011) as "a compact area of at least 300 population or about 60–70 households of poorly built congested tenements, in unhygienic environment usually with inadequate infrastructure and lacking in proper sanitary and drinking water facilities" [36]. We used this definition for all practical purposes in this study.

### Research design

We used a cross-sectional ecologic study design to explore the relationship between tobacco-related built environment distribution and tobacco consumption prevalence. This study was conducted in randomly selected 32 urban slums of Bhopal city. The study unit was a "slum cluster", a geographically demarcated area (by identifiable structures like roads, water bodies or boundary walls, etc.) covering 100 to 150 consecutive households within each selected slum area. Thus, a total of 32 such clusters were identified in 32 selected (one from each) slum areas.

### Survey development

**Fig. 1:**
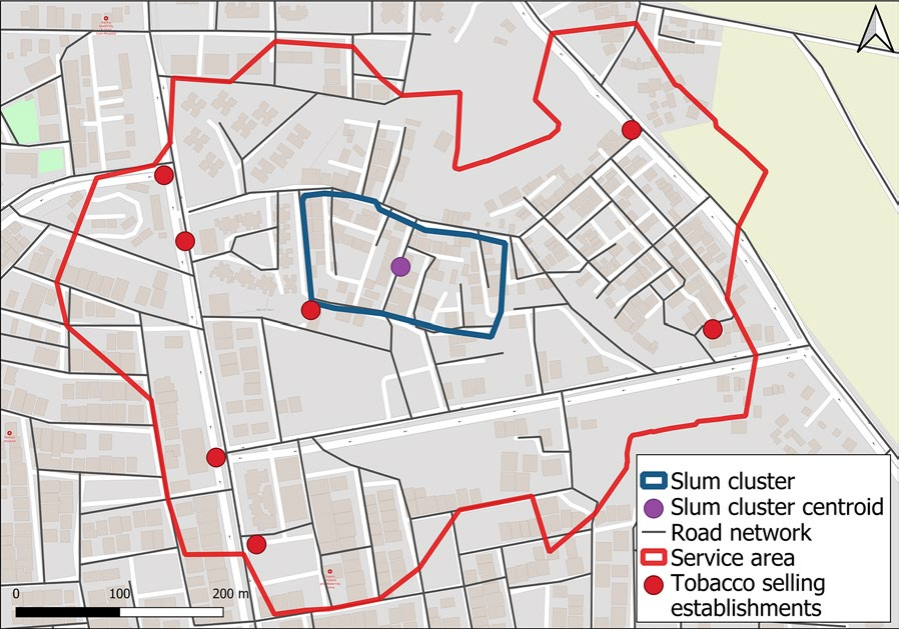
Distribution of tobacco shops in the neighbourhood (400 m service area) of one slum cluster

GIS was used for mapping the distribution of tobacco-related built environment in the neighbourhood of slum clusters. 'Neighbourhood or service area' was operationally defined as an area equivalent to a 5 to 15 min walk, i.e., 400 m in any direction by road from the centroid of a slum cluster [37]. Centroids of the slum clusters were plotted using the 'Centroids' function under the 'geometry' tool under the 'vector' tab in QGIS software version 2.18.3. To plot the neighbourhood geographically, we used the 'make service area layer' analysis under the 'network analyst' tool in the ArcMap software version 10.7.1[38, 39]. (Fig. 1).

### Data collection and processing

#### Mapping neighbourhood or slum cluster service area

The investigators randomly identified a cluster of about 100 to 150 geographically adjoining households within the selected slum. Geographic boundaries of the chosen slum cluster were plotted using identifiable structures like roads, water bodies or boundary walls, etc., by two field workers using open access, mobile phone-based survey platforms (https://ona.io with GeoODK app). The plotted boundaries of all slum clusters were subsequently imported into GIS and saved as shape (.shp) files. The map sheet was registered on the Earth's surface using the geo-referencing process with suitable projection for the study area's geographic coordinates, i.e., Bhopal, MP, India (Selected CRS EPSG:32644, WGS 84/UTM zone 44N). Validation of the data was done by matching point locations of certain identifiable features (such as crossroads, water bodies, large buildings, etc.) obtained from hand-held GPS equipment (Garmin Map64) with the locations of corresponding identifiable features in the base maps (Namely; Topographic, Streets and Imagery) available in the ArcMap software version 10.7.1. A service area of 400 m was plotted for each slum cluster using the 'make service area layer' analysis under the 'network analyst' tool in the ArcMap software version 10.7.1 [38, 39]. 'Service area maps' with identifiable features were prepared and provided to the field workers for mapping and data collection of tobacco shops. [38, 40]. (Fig. 1).

#### Operational definitions

In India, every state (province) has its own shop and establishment act (law) [41]. Under the act, shop is defined as "a premise where the selling of goods take place either by retail or wholesale or where services are rendered to customers" [42]. This act regulates all the shops operating in the state by issuing licences through a formal registration process. Operationally, in this study, the shops involved in the retail sale of tobacco products were identified as 'tobacco shops' to study tobacco-related built environment. Although registration is mandatory for all shops, many tobacco shop owners manage to run their shops without any licence. However, the licenced shopkeepers must display their licences in a visible place in their shop. In this study, our survey team observed these licenses to classify tobacco shops as 'organised' or 'unorganised' based on the availability or non-availability of shop and establishment act licences, respectively.

For analysis, we classified tobacco shops as 'exclusive tobacco shops' or 'other tobacco shops'. Exclusive tobacco shops mainly sell tobacco and related products, whereas 'other tobacco shops' are primarily outlets of some other products (like grocery) but also sell tobacco products. We operationally defined a current tobacco user as "a person who used any tobacco product in last 30 days (smoked or smokeless form or both forms, daily or non-daily basis, irrespective of the quantity)" [4, 43–45].

#### Fieldwork

To estimate the prevalence of tobacco use, individuals aged more than 30 years (excluding pregnant women) residing in the selected slum clusters for at least the last 6 months were enrolled after obtaining written informed consent. Two trained field workers administered a semi-structured questionnaire to collect information regarding tobacco use and socio-demographic variables (age, gender, literacy and household asset ownership data), which were documented to be associated with the tobacco use prevalence. [46–48].

Wealth index was calculated by principal component analysis of household assets (i.e. number of consumer items owned by the household, such as television, radio, fridge, car, sanitary facilities, floor material and other items related to wealth). The asset scores were standardised by relating them with the standard normal distribution of a mean of zero and a standard deviation of one. A higher wealth index score indicates a relatively better socioeconomic position. Detailed information on the calculation of the wealth index scores is provided elsewhere [49].

Surveyors identified and visited all tobacco shops within the 400 m service area of the slum clusters by transect walk method with the help of 'service area maps' described in the previous mapping section. Closed tobacco shops were revisited and were dropped from the study if no contact could be made after repeated visits. The survey team collected relevant details of the tobacco shops (like floor area, structure, availability of shop and establishment act license, etc.) and geographic coordinates using a semi-structured questionnaire on mobile phone-based survey platforms.

### Statistical analysis

ArcMap (version10.7.1) and QGIS software (version 3.20.0) were used for analysing spatial data [38, 40]. All other bio-statistical analysis was done in SPSS version 25 software [50]. Descriptive categorical data were presented as numbers, percentages and frequency tables. Descriptive numerical data were displayed as mean (and standard deviation) or median (and Inter Quartile Range, IQR) depending on the distribution of data (normal or skewed, respectively). The number of tobacco shops in a service area of each slum cluster was calculated using the 'count points in a polygon' function of the 'analysis' tool in the 'vector' tab of QGIS 3.20.0. The area of the cluster neighbourhood (service area) was calculated using the '$area' function in the 'field calculator' tab of QGIS 3.20.0. The density of tobacco shops in the neighbourhood of each slum cluster was calculated by dividing the number of tobacco shops in the neighbourhood of a particular slum cluster by the area of the corresponding neighbourhood. Descriptive spatial data was presented as the density of tobacco shops per square kilometre (/km^2^).

Multiple linear regression (MLR) analysis was performed to examine the relationships between tobacco use prevalence in slum clusters and the density of tobacco shops (all, exclusive and other tobacco shops separately). Analyses were adjusted for documented demographic and socioeconomic variables of the study participants residing in the clusters (i.e., cluster aggregate values for age, literacy, wealth index and gender ratio) [46–48]. While building models, multicollinearity was tested in between included variables (multicollinearity was ruled out if variance inflation factor or VIF < 5) [51, 52].

## Results

### Demographic characteristics of the study population and tobacco use

We conducted a tobacco use prevalence survey on 6214 eligible and consenting adults above 30 years of age in 32 slum clusters in Bhopal. Among them, 3489 (56.1%) were females, and 2725 (43.9%) were males. The prevalence of current tobacco use among all 32 slum clusters ranged from 22.1 to 59.6% (median 40.9% with IQR 31.8–44.2) (Table 1).

**Table 1:** Demographic profile of the participants and descriptive statistics of the tobacco-related built environment

Slum cluster serial no.	Adult respondent (N)	Age Mean (SD)	Females N (%)	Males N (%)	Tobacco users N (%)	The density of tobacco shops in service area (exclusive and other) N/km^2^	Mean Wealth Index	Literate population (%)
1	143	45.3 (13.2)	85 (59.4)	58 (40.6)	61 (42.7)	44.89	− 0.48	58.57
2	188	43.1 (10.1)	114 (60.6)	74 (39.4)	79 (42)	46.04	− 0.70	58.51
3	226	44.8 (12.8)	119 (52.7)	107 (47.3)	65 (28.8)	49.70	0.98	78.92
4	307	43.4 (12.3)	172 (56)	135 (44)	68 (22.1)	61.68	0.87	85.02
5	185	43.2 (11.5)	86 (46.5)	99 (53.5)	54 (29.2)	183.49	0.61	87.10
6	264	43.8 (13.8)	137 (51.9)	127 (48.1)	97 (36.7)	155.44	0.21	66.67
7	215	45.6 (12.9)	109 (50.7)	106 (49.3)	107 (49.8)	226.61	0.46	71.51
8	324	46.5 (14.5)	181 (55.9)	143 (44.1)	139 (42.9)	70.25	− 0.24	59.70
9	209	45 (11.8)	108 (51.7)	101 (48.3)	85 (40.7)	195.10	0.63	81.03
10	266	44.1 (12.7)	138 (51.9)	128 (48.1)	83 (31.2)	153.72	0.63	80.16
11	125	42 (9.8)	69 (55.2)	56 (44.8)	66 (52.8)	149.08	− 1.02	66.92
12	197	42.8 (10.3)	106 (53.8)	91 (46.2)	84 (42.6)	109.29	− 0.57	70.32
13	101	39.9 (8.7)	60 (59.4)	41 (40.6)	45 (44.6)	107.71	− 1.20	64.00
14	161	39.8 (8.6)	80 (49.7)	81 (50.3)	71 (44.1)	138.75	− 1.40	64.41
15	171	45.6 (12.3)	88 (51.5)	83 (48.5)	82 (48)	53.87	0.23	62.94
16	219	45.1 (12.3)	134 (61.2)	85 (38.8)	97 (44.3)	53.87	0.09	55.25
17	158	43.5 (9.9)	89 (56.3)	69 (43.7)	65 (41.1)	71.64	0.14	81.01
18	231	43.4 (10.5)	144 (62.3)	87 (37.7)	86 (37.2)	57.12	0.28	86.96
19	160	45.9 (12.4)	83 (51.9)	77 (48.1)	92 (57.5)	90.77	0.05	53.16
20	258	47.9 (11.8)	137 (53.1)	121 (46.9)	154 (59.7)	69.44	− 0.03	45.42
21	153	45.3 (11.4)	79 (51.6)	74 (48.4)	59 (38.6)	37.82	0.37	52.31
22	186	46.2 (12.7)	100 (53.8)	86 (46.2)	80 (43)	34.01	0.23	49.74
23	176	46.3 (14.5)	104 (59.1)	72 (40.9)	59 (33.5)	32.21	0.41	76.58
24	259	45.4 (12.6)	154 (59.5)	105 (40.5)	93 (35.9)	40.58	0.51	73.84
25	185	46.9 (14.6)	108 (58.4)	77 (41.6)	45 (24.3)	40.69	0.95	81.11
26	219	44.5 (12.6)	136 (62.1)	83 (37.9)	61 (27.9)	32.83	0.85	82.03
27	159	44 (10.5)	89 (56)	70 (44)	48 (30.2)	31.40	− 0.17	70.27
28	110	44.3 (11.4)	80 (72.7)	30 (27.3)	38 (34.5)	31.60	− 0.06	61.26
29	135	43.7 (11.1)	84 (62.2)	51 (37.8)	46 (34.1)	68.93	0.52	51.13
30	170	44.5 (12.1)	106 (62.4)	64 (37.6)	78 (45.9)	67.78	− 0.27	54.78
31	178	44.2 (12.7)	102 (57.3)	76 (42.7)	47 (26.4)	20.46	− 0.22	66.06
32	176	44.4 (12.2)	108 (61.4)	68 (38.6)	74 (42)	17.83	− 0.32	45.45
Total	6214	44.55 (12.27)	3489 (56.1)	2725 (43.8)	2408 (38.7)	59.40 (IQR 39.9–108.1)	0.074 (SD 0.61)	66.9 (SD 12.5)

### Descriptive information on the tobacco-related built environment

We found 194 tobacco shops in the service area of all the clusters. Among them, only 35 (18%) of tobacco shops belonged to the organised sector (had shop and establishment act licence). Only 42 (21.7%) were exclusive tobacco shops, whereas 152 (78.3%) were other tobacco shops. Most (140, 72.2%) tobacco shops had a permanent fixed structure. The 'No smoking' sign was visible in only two (1%) tobacco shops. A very high number of tobacco shops (160, 82.5%) were displaying tobacco products openly. We found that only two (1%) tobacco shops displayed a sign of "age 18 years and below not allowed to buy tobacco". Another critical observation of the current study is that a substantial percentage of tobacco shops (93, 47.9%) were situated within a 100-m radius of educational institutions.

The median number of tobacco shops in the service areas of clusters was 12 (IQR 5–26), mean area of service areas around clusters was 0.19 km (SD 0.038 km). The median density (unit: tobacco shops/km^2^) of tobacco shops in service areas around slum clusters was 59.4 (IQR 39.9–108.1)/km^2^ (Table 1).

### Association between tobacco shop density and tobacco use prevalence

Tobacco use prevalence was significantly associated with the density of all tobacco shops (estimate or B = 0.071, *p* value < 0.002) and other tobacco shops (estimate or B = 0.058, *p* value 0.010) after adjusting for age, literacy and wealth index. The association between tobacco use prevalence and the density of exclusive tobacco shops was not significant (estimate or B = 0.207, *p* value 0.116) (Table 2).

**Table 2:** Multiple linear regression (MLR) models for tobacco use prevalence

Final models of MLR	Estimate (B)	Standard error of estimate (SE B)	*p* value	Adjusted R^2^ of model
**For all tobacco shops**				0.656
Intercept	− 72.905	43.766	0.108	
Density of all tobacco shops (No./km^2^)	0.071	0.021	0.002	
Age in years (mean)	2.740	0.857	0.004	
Percentage of literate population	− 0.250	0.113	0.035	
Wealth Index (mean)	− 9.645	2.711	0.001	
Gender ratio (percent male population)	0.048	0.223	0.831	
**For other tobacco shops**				0.614
Intercept	− 74.038	46.353	0.122	
Density of other ﻿tobacco shops (No./km^2^)	0.058	0.021	0.010	
Age in years (mean)	2.716	0.908	0.006	
Percentage of literate population	− 0.229	0.119	0.064	
Wealth Index (mean)	− 10.049	2.863	0.002	
Gender ratio (percent male population)	0.108	0.235	0.648	
**For exclusive tobacco shops**				0.547
Intercept	− 82.940	50.153	0.110	
Density of exclusive ﻿tobacco shops (No. per km^2^)	0.207	0.127	0.116	
Age in years (mean)	2.534	0.982	0.016	
Percentage of literate population	− 0.159	0.125	0.216	
Wealth Index (mean)	− 9.704	3.139	0.005	
Gender ratio (percent male population)	0.417	0.228	0.079	

Dependent variable: tobacco use prevalence (current) in slum clusters

### Association between other risk factors and tobacco use prevalence

Tobacco use prevalence was also significantly associated with mean age and mean wealth index in all the models. The percentage of literate population was significantly associated with the prevalence of tobacco use in the model for all tobacco shops. Gender ratio was not associated with tobacco use prevalence in any of the models. We have not found any multicollinearity (VIF < 5) in between variables included in any of the models (Table 2).

## Discussion

We used GIS-based methods in this study to establish the tobacco-related built environment in 32 urban slum clusters of Bhopal. We found that the median density of tobacco shops was 59.40 (IQR 39.9–108.1)/km^2^ in the urban slums of Bhopal. Another study conducted in North India found that the median density of tobacco stores was 82.9/km^2^ in rural areas and 34.6 (9.0–91.0)/km^2^ in urban colonies [19]. Loomis et al. reported a density of 5.1/km^2^ in New York, while another study conducted in New York by Hyland et al. reported a density of 1.2 to 4 per 10 km of street length [53, 54]. Lower densities reported by studies done in New York could be because these studies reported only licensed tobacco retailers. In our study, the proportion of tobacco outlets in the organised sector (having shop and establishment act licence) was only 18% of the total tobacco-related built environment.

In the current research, tobacco use prevalence among the study population (≥ 30 years of age) was 38.7% (ranging from 22.1 to 59.6% among 32 clusters), which was similar to the tobacco use prevalence (38.4%) in the population above 25 years reported in India in Global Adult Tobacco Survey-2 (2016–2017) [5, 55].

In our research, we found that tobacco use prevalence significantly rises with higher age, lower wealth index and lower literacy rates. Similar results have also been obtained in previous nationwide surveys conducted in India. Age is an established risk factor for tobacco use because older adults are independent in deciding to consume tobacco. It has also been documented that people with higher socioeconomic strata and better literacy rates are less likely to consume tobacco on the account of their better health awareness. In ecological studies, gender ratio plays an important role in tobacco consumption because higher prevalence in males has been reported by previous surveys conducted in India [4, 43, 46].

The tobacco use prevalence among adults in the selected slum clusters was significantly associated with the density of tobacco shops (all and other) after adjusting for known confounders (Table 2). Non-significant association with exclusive tobacco shops could be because of the smaller number (N = 42) of such establishments. A recently published study by Golden et al. presenting country-level analysis from the United States of America (USA) reported that the association between smoking prevalence and tobacco retailer density was statistically significant in metropolitan counties [27]. Johns et al. reported that tobacco store density was associated with tobacco consumption (Odds Ratio: 1.41) [24]. Henricksen et al. reported that high school smoking prevalence was associated with the density of tobacco outlets and tobacco advertising in school neighbourhoods [25]. A study conducted in 20 North Indian communities documented that tobacco shop densities were significantly associated (OR: 1.8; 95% CI 1.1–3.3) with tobacco consumption in multivariate analysis [19]. Lee et al., in their recently published systematic review and meta-analysis of 27 studies, documented an estimated 2.48% reduction in risk of tobacco use with reduced exposure to tobacco retailer density and proximity [23].

Various indirect pieces of evidence have also suggested the causality of such associations. A cohort study from the USA reported that the high tobacco outlet density was associated with the recent initiation of tobacco use among adults in the neighbourhood [28]. A high density of tobacco shops in an area might increase the ease for an individual to access tobacco products, which further leads to local acceptability and normalisation of tobacco-using behaviours. Such behaviours could collectively affect the prevalence of tobacco use in that locality [56]. A longitudinal study designed to study the efficacy of a smoking cessation program in Dallas (Texas, USA) reported that the proximity of a participant's home to a tobacco outlet (less than 1 mile) was significantly associated with stronger smoking urges among smokers trying to quit [22]. Few research studies, which explored the association between distance from tobacco shops and attempts to quit smoking, reported that proximity to tobacco retailers reduces the chance of smoking cessation [26, 57]. Research has also found that the sight of tobacco retail outlets is associated with tobacco purchase and increased frequency of its use [29].

A higher proportion of other tobacco shops (78.3%), as compared to exclusive tobacco shops (21.7%) in the current study, suggests that there could be high demand for tobacco in the community. Increased demand for tobacco products might also increase the opening of new tobacco retail shops and escalate the situation into a vicious cycle. A high density of tobacco shops in a particular locality may trigger this vicious cycle, further resulting in a worsening tobacco situation in that community [23, 56].

All these considerations indicate that tobacco shop density is a significant environmental risk factor in the epidemiology of tobacco use. But estimation of tobacco shop density could be challenging in settings where reliable secondary data on tobacco shops are unavailable. This study provides a feasible but robust methodology to generate original data on the tobacco-related built environment, which is essential to form context-specific tobacco control strategies.

We also observed that the tobacco shops in the study area were violating COTPA 2003 guidelines, like the absence of a "no smoking sign", the lack of any signage prohibiting the sale of tobacco to those under 18 years and the location of tobacco shops within 100 m of schools. This may be because tobacco was mainly sold by the unorganised sector (82%), which is difficult to put under a legal framework. A high proportion of shops in the unorganised sector, i.e. shops not registered under the shops and establishment act, could be due to lack of awareness among the shop owners because of their low level of education. Moreover, a relatively low investment cost is required to establish a tobacco shop. Therefore, tobacco shop owners do not have much to lose, even in the case of legal action requiring the shop's closure and the prohibition of the sale of its goods. This issue can be addressed by implementing relevant policy changes to ensure the registration of all tobacco shops under the shop and establishment act. Policymakers can also utilise this process to ensure compliance of these shops with COTPA 2003 guidelines. The licensing process should also consider tobacco shop density in a locality while issuing new licenses. Based on these findings, we recommend regulating the tobacco-related built environment through mandatory licensing of all tobacco shops.

The density of tobacco shops is an important quantifiable characteristic of the tobacco-related built environment. One of the study's strengths is that it used original data to estimate the density of tobacco shops in the service area (that includes all the streets that can be reached within 400 m from cluster centroid) using GIS tools like the 'network analysis'. However, the study's findings should be interpreted in light of its limitations, such as (1) the study's cross-sectional nature did not allow us to predict the direction of the association. (2) As the study was conducted in the urban slums of Bhopal city, its findings may not be genralisable to other settings. (3) This research was a part of another study designed to explore non-communicable disease (NCD) related risk factors among the adult (> 30 years) population of urban slums of Bhopal city. Due to this, we could not study the effect of the tobacco-related built environment on the adolescent and young adult population, who are also likely to be influenced by the neighbourhood built environment. These limitations could be overcome by conducting a longitudinal study with a larger sample size and including participants below 30 years. Further research is also required to study the impact of licensing process on tobacco shop density and tobacco use prevalence.

## Conclusions

Tobacco shop density was significantly associated with tobacco use prevalence after adjusting for socio-demographic variables. Compliance with the COTPA guidelines was poor in most of the surveyed tobacco shops. Appropriate policy interventions are required to regulate tobacco-related built environment to control rampant tobacco use.

## Data Availability

The datasets used and/or analysed in the current study are available from the corresponding author upon reasonable request.
